# Sexual dysfunction induced by psychotropic drugs: a narrative review.

**DOI:** 10.1192/j.eurpsy.2023.2328

**Published:** 2023-07-19

**Authors:** C. Álvarez

**Affiliations:** Hospital Universitario Príncipe de Asturias, Madrid, Spain

## Abstract

**Introduction:**

One fairly common side effect of psychopharmaceuticals is sexual dysfunction. They can influence various aspects of sexual function, including lubrication, desire, ejaculation, and orgasmic intensity… This may worsen mental health conditions and make it challenging for patients to adhere to the Treatment. We’ll examine how these drugs affect the area of sexual activity.

**Objectives:**

To emphasise how different antipsychotics and antidepressants may affect sexual function.

**Methods:**

We conducted a narrative review about the available literature on the subject. Articles were selected based on their clinical relevance.

**Results:**

All SSRI antidepressants carry a considerable risk of sexual dysfunction. According to some research, escitalopram and paroxetine may pose the greatest risk among this group. Similar risks of sexual issues exist with SNRIs. Bupropion, on the other hand, has a lot of evidence demonstrating low or no risk. Agomelatine, mirtazapine, and moclobemide also have a minor impact on sexual performance.

Hyperprolactinemia has been specifically linked to sexual impairment in antipsychotic medication, hence antipsychotics that cause hyperprolactinemia such as haloperidol, risperidone, paliperidone, and amisulpride are more likely to induce sexual disturbances. Aripiprazole, quetiapine, and ziprasidone have been demonstrated to be less or not connected to sexual dysfunction.

**Conclusions:**

Although sexual dysfunction is not an unusual side effect of psychiatric medications, it is frequently underdiagnosed. Sexuality needs to be explored by therapists because it may affect patient’s treatment compliance and well-being. It’s critical to understand how each psychotropic drugs can impair sexual function in order to select the best option based on the individual traits of each person.

**Disclosure of Interest:**

None Declared

